# Genetic toxicology and toxicogenomic analysis of three cigarette smoke condensates in vitro reveals few differences among full-flavor, blonde, and light products

**DOI:** 10.1002/em.21689

**Published:** 2012-03-19

**Authors:** Carole L Yauk, Andrew Williams, Julie K Buick, Guosheng Chen, Rebecca M Maertens, Sabina Halappanavar, Paul A White

**Affiliations:** Environmental Health Science and Research Bureau, Health CanadaOttawa, Ontario, Canada

**Keywords:** gene expression, DNA mutation, micronucleus, bronchial epithelial cells, toxicogenomics, DNA microarrays

## Abstract

Cigarette smoking leads to various detrimental health outcomes. Tobacco companies produce different brands of cigarettes that are marketed as reduced harm tobacco products. Early examples included “light” cigarettes, which differ from regular cigarettes due to filter ventilation and/or differences in chemical constituents. In order to establish baseline similarities and differences among different tobacco brands available in Canada, the present study examined the cytotoxicity, mutagenicity, clastogenicity, and gene expression profiles of cigarette smoke condensate (CSC) from three tobacco products, encompassing a full-flavor, blonde, and “light” variety. Using the Salmonella mutagenicity assay, we confirmed that the three CSCs are mutagenic, and that the potency is related to the presence of aromatic amines. Using the Muta™Mouse FE1 cell line we determined that the CSCs were clastogenic and cytotoxic, but nonmutagenic, and the results showed few differences in potencies among the three brands. There were no clear brand-specific changes in gene expression; each brand yielded highly similar expression profiles within a time point and concentration. The molecular pathways and biological functions affected by exposure included xenobiotic metabolism, oxidative stress, DNA damage response, cell cycle arrest and apoptosis, as well as inflammation. Thus, there was no appreciable difference in toxicity or gene expression profiles between regular brands and products marketed as “light,” and hence no evidence of reduced harm. The work establishes baseline CSC cytotoxicity, mutagenicity, and expression profiles that can be used as a point of reference for comparison with data generated for products marketed as reduced harm and/or modified risk tobacco products. Mol. Mutagen. 2012. © 2012 Wiley Periodicals, Inc.†

## INTRODUCTION

Cigarette smoking constitutes a major global human health hazard. Smoking leads to a host of detrimental health outcomes including cancer [IARC,^2004^] and cardiovascular disease [Burns,^2003^]. Tobacco smoke contains over 4,000 chemicals, including chemicals that are both mutagenic and carcinogenic [reviewed in Hecht,^1999^; DeMarini,^2004^]. Indeed, there are over 70 known carcinogens in mainstream tobacco smoke [Hecht,^2012^].

Tobacco companies have introduced a variety of cigarette products, including light cigarettes, and more recently, cigarettes with novel filters containing activated charcoal, which are collectively referred to as reduced harm tobacco products. These products are marketed to people who hope to reduce the detrimental health effects associated with smoking. Products labeled as “light” cigarettes, a designation that has been voluntarily removed in Canada since 2001, may have been viewed by consumers as less addictive or toxic than full-flavor varieties. Ventilation holes in the filters of “light” cigarettes were designed to provide the impression that the smoker is experiencing a reduction in the exposure to tobacco smoke and its constituents. However, it has become apparent that existing reduced harm tobacco products, such as brands formerly marketed as “light,” present no obvious reduction in harm compared with regular, full flavor products [Hecht et al.,^2005^]. Moreover, it is now clear that smokers compensate for increased filter ventilation in “light” cigarettes by modifying their puffing behavior [Rickert and Robinson,^1981^; Kabat,^2003^; Benowitz et al.,^2005^; Hammond et al.,^2005^]. Modifications include stronger puffing (i.e., larger and more frequent) that potentially results in higher nicotine, tar, and carbon monoxide extraction. Furthermore, it has been suggested that changes in smoking behavior (e.g., stronger puffs) contribute to increases in carcinogen exposure (per cigarette), as well as changes in the concentrations of other smoke constituents [Thornton^1966^; Schneider^1992^; Otmar and Kotzias^2007^].

Commercial cigarette types vary with respect to the chemical composition of the inhaled smoke [Chepiga et al.,^2000^], with differences not only in tar and nicotine, but also in various chemical families including: polycyclic aromatic hydrocarbons (PAHs) [Ding et al.,^2005^], tobacco-specific nitrosamines (TSNAs) [Ashley et al.,^2003^], phenols [Vaughan et al.,^2008^], and metals [Pappas et al.,^2007^; Hammond and O'Connor,^2008^]. Chemical composition of mainstream smoke is also dependent on the various additives used, the paper type, the tobacco processing, and other manufacturer-specific features [Otmar and Kotzias,^2007^].

In addition to differences in chemical composition, the various tobacco brands also exhibit differences in toxicity. For example, Bernfeld [1975] exposed female CAF1/J mice to whole smoke, or its gas phase, and found marked differences in the acute toxicities of whole smoke across 10 brands. Ritter et al. [2004] demonstrated statistically significant differences in glutathione depletion induced in a human type II-like lung cell line following exposures to whole smoke and filtered smoke from three cigarette types. A few studies have examined mutagenic activity across various brands relative to a reference tobacco smoke condensate [Chepiga et al.^2000^; Foy et al.,^2004^]. For example, Foy et al. [2004], Chepiga et al. [2000] and Doolittle et al. [1990] employed the Salmonella mutagenicity assay to document differences in the mutagenicity of cigarette smoke condensates (CSC) across various products, including full flavor, low tar, and ultra-low tar brands. These studies revealed relatively small differences in the mutagenic activity of CSCs (as revertants per unit tar or TPM) representing the variety of products marketed in the United States. Two previous studies have used global transcriptomic analyses to establish brand-specific toxicogenomic profiles [Lu et al.,^2007^; Pickett et al.,^2010^]. These authors suggest that expression signatures can be used to distinguish certain brands or types of cigarettes.

In the present study, we employed the Salmonella reverse mutation assay and the in vitro *LacZ* transgene mutation assay in Muta™Mouse FE1 cells [White et al.,^2003^] to evaluate the mutagenicity of CSC samples from three cigarette brands representing three tobacco products available in Canada. Muta™Mouse FE1 cells were also employed to assess cytotoxicity via clonogenic survival and clastogenicity via the cytokinesis-block micronucleus assay [Fenech,^2005^]. In addition, a comprehensive analysis of gene expression changes in FE1 cells exposed to CSC was also conducted. The work had three primary objectives: (1) to characterize the toxicity and mutagenicity of three representative Canadian tobacco products; (2) to employ gene expression profiling in parallel with cytotoxicity and mutagenicity to provide a better understanding of the mechanism of action of CSC and shed light on any distinct toxicological mechanisms underlying the in vitro effects observed for different tobacco products; and (3) to provide a baseline profile of mutagenicity, cytotoxicity, and gene expression changes against which new claims for modified risk tobacco products can be evaluated.

## MATERIALS AND METHODS

### Cigarette Smoke Condensate

CSC were prepared for three popular Canadian commercial tobacco products referred to hereafter as Brand 1, Brand 3, and Brand 5. In the remainder of the manuscript the term “brand” is used to refer to a tobacco product currently or formerly available in the Canadian market. Brand 1 is a nonventilated Virginia flue-cured product that is marketed as a full-flavor cigarette. Brand 3 contains a mixture of tobacco types and is marketed by the manufacturer as “blonde.” Brand 5 contains Virginia flue-cured tobacco and is marketed by the manufacturer as “light.” All three brands are filtered cigarettes; Brands 3 and 5 contain ventilated filters. Cigarettes were smoked and CSC samples were prepared by Labstat International Inc. (Kitchener, Ontario) as previously described [Moir et al.,^2008^]. Briefly, each of the three cigarette brands was smoked in triplicate using a Borgwaldt 20-port rotary smoking machine following International Organization for Standardization (ISO) standard 3308 (i.e., puff volume of 35 mL, puff duration of 2 sec, puff interval of 60 sec). The smoke was passed through a 92-mm glass fiber filter disk for particulate matter collection according to the Health Canada official test method [Health Canada,^2004^]. The number of cigarettes smoked for each brand and the total yield of particulate matter (TPM) are provided in Table I. To prepare condensate samples, filter pads were placed in a flask containing dimethyl sulfoxide (DMSO) (ACS spectrophotometric grade, >99.9%) and shaken on a wrist-action shaker (model 3589, Barnstead International) for 20 min. Each sample was standardized to a concentration of 30 mg TPM per mL of DMSO.

**TABLE I:** Number of Cigarettes Smoked and TPM Yield for each CSC. Each Condensate was Prepared in DMSO, and Standardized to a Concentration of 30 mg TPM/mL

Brand	Tobacco type	Filter	Brand designation^a^	Total no. cigarettes smoked	TPM yield (mg)	TPM/cigarette
1	Virginia flue-cured	Yes, no ventilation	Full-flavor	60	1,625.5	27.09
3	Mixed	Yes, ventilation	Blonde King size	108	1,826.0	16.91
5	Virginia flue-cured	Yes, ventilation	Light King size	117	1,659.0	14.18

a Manufacturer designation. Blonde refers to a light-colored mixed tobacco that is common in US cigarettes. Full-flavor brands are those that are not marketed as light.

### Salmonella Mutagenicity Assay

CSC samples were tested for mutagenic activity using the preincubation version of the Salmonella mutagenicity assay as described in Mortelmans and Zeiger [2000]. Briefly, CSC were combined with the Salmonella tester strain, a metabolic activation mixture derived from Aroclor 1254-induced rat liver, and incubated for 20 min at 37°C. The contents were then mixed with molten agar and poured onto glucose minimal media agar plates. Seven concentrations of each of the CSC triplicates, ranging from 3 to 250 μg TPM per plate, were tested depending on the potency of the sample on a given Salmonella strain (i.e., three brands, three replicate CSC per brand, tested at seven concentrations each alongside solvent control). Each concentration was tested in triplicate. Plates were inverted and incubated at 37°C for 72 hr. Following incubation, the number of revertant colonies on each plate was scored using a Protocol RGB Colony Counter (Synbiosis). Three bacterial test strains were used, including the standard frameshift tester strain TA98, as well as two metabolically enhanced versions of TA98 known as YG1041 and YG5161. YG1041 overexpresses the Salmonella classical nitroreductase and *O*-acetyl transferase enzymes, and shows enhanced sensitivity to nitroarenes and aromatic amines [Hagiwara et al.,^1993^]. YG5161 overexpresses the *dinB* gene, encoding *Escherichia coli* DNA polymerase IV, and shows enhanced sensitivity to unsubstituted PAHs [Matsui et al.,^2006^]. Strains YG1041 and YG5161 were obtained directly from Dr. Takehiko Nohmi (National Institute of Health Sciences, Japan). Preliminary testing showed a lack of response without exogenous metabolic activation (mean responses to positive controls were 450 ± 94 rev/plate ± SD for 0.5 μg/plate daunomycin and 736 ± 67 rev/plate ± SD for 3 μg/plate 2NF for TA98 and YG1041, respectively). Samples were therefore tested in the presence of a mixture containing postmitochondrial supernatant (S9) derived from Aroclor 1254-induced rat liver. The S9 metabolic activation mixture consisted of 2% (v/v) microsomal salt solution (0.4 M MgCl_2_ and 1.65 M KCl), 5 mM glucose-6-phosphate monosodium salt (Sigma-Aldrich), 4 mM NADP disodium salt, in 0.1 M phosphate buffer pH 7.4 with 5% (v/v) Aroclor 1254-induced rat liver S9 (Moltox Inc.). Protein levels were 35.7 to 43.5 mg/mL of rat liver S9, resulting in 0.9 to 1.1 mg of S9 protein per plate; 2-aminoanthracene (2AA) was employed as the positive control to ensure assay performance. All Salmonella mutagenicity data are available from the corresponding author on request.

### FE1 Cell Culture

The FE1 cell line, which was derived from Muta™Mouse lung epithelium, was cultured as described in White et al. [2003]. Baseline gene expression characteristics of cultured FE1 cells at confluence and subconfluence are described in Berndt-Weis et al. [2009]. Briefly, cells were cultured in 1:1 DMEM:F12 nutrient mixture supplemented with 2% FBS, 2 mM glutamine, 100 U/mL penicillin G, 100 μg/mL streptomycin sulphate, and 1 ng/mL murine epidermal growth factor (Invitrogen Life Technologies, Canada). Incubations were conducted at 37°C, 95% humidity, and 5% CO_2_. Confluence (%) was determined using replicate plate counts. Cells on replicate plates were trypsinized and aliquots used to assess cell number using a Coulter Particle Counter (Beckman Coulter). This measurement was compared with a predetermined cell count for a completely confluent plate (100%). Cells from three replicate plates at 50% confluence were collected for RNA isolation.

### Cytotoxicity Assessment

Cytotoxicity of CSC samples was determined using a clonogenic survival assay. Briefly, 2 to 3 × 10^5^ FE1 cells were seeded at approximately 20% confluence on 100 mm polystyrene culture plates and incubated overnight. Plates were counted to determine cell density, and duplicate plates at a known cell density were exposed, in triplicate, for 6 hr in serum-free medium to 0, 30, 60, 90, and 120 μg TPM/mL media of each of the three CSCs. Following the exposure, cells were rinsed with PBS, trypsin treated, removed from the plate, counted, appropriately diluted, and plated on triplicate plates for colony formation (7–10 days). Plates were then rinsed with PBS, colonies fixed by treatment for 5 min with 90% methanol, and stained with Giemsa (1:10 dilution of KaryoMAX Giemsa, Invitrogen, Canada). Cells exposed to the solvent alone (i.e., DMSO) showed a mean colony forming efficiency of 19.4 ± 1.4%.

### Muta™Mouse LacZ Transgene Mutation Assay

Cells were treated as described in White et al. [2003]. Briefly, 2 to 3 × 10^5^ FE1 cells were seeded on 100 mm polystyrene culture plates, incubated overnight to approximately 20% confluence, and exposed to a range of CSC concentrations (10–200 μg/mL) for 6 hr in serum-free medium. Since FE1 cells are known to express cytochrome p450 1A1 (*Cyp1A1*), and are capable of activating mutagenic carcinogens such as benzo[*a*]pyrene (BaP) [White et al.^2003^], initial assessment was conducted in the absence of any exogenous activation mixture. Subsequent assays employed an S9 metabolic activation mixture containing 0.5, 1, 2, or 4% (v/v) Aroclor-induced rat liver S9. In addition, selected assays employed preincubation of the CSC with the S9 mixture for 15, 30, or 60 min at 37°C. Positive controls included 0.4 μM BaP without exogenous activation and 2 μM PhIP (2-amino-1-methyl-6-phenylimidazo[4,5-*b*]pyridine) with exogenous S9 activation.

DNA isolation and scoring of transgene mutant frequency was carried out as described [Gossen and Vijg,^1993^; Vijg and Douglas,^1996^; White et al.^2003^]. Briefly, exposed FE1 cells were rinsed with PBS and digested overnight in lysis buffer containing 10 mM Tris pH 7.6, 10 mM EDTA, 10 mM NaCl, 1 mg/mL proteinase K, and 1% SDS. DNA was isolated and purified using chloroform/phenol extraction and precipitation in ethanol. Freshly isolated DNA was dissolved in Tris-EDTA buffer and stored at 4°C until scoring. Transgene mutant frequency was determined using the phenyl-β-d-galactopyranoside (P-gal) positive selection assay. Briefly, λgt10 *lac*Z DNA copies were rescued from genomic Muta™Mouse DNA using the Transpack™ lambda packaging system (Stratagene). Packaged phage particles were mixed with host bacterium (*E. coli* Δ*lac*Z, *gal*E^−^, *rec*A^−^, pAA119 with *galT* and *galK*) [Gossen and Vijg,^1993^; Vijg and Douglas,^1996^], plated on minimal agar with 0.3% w/v P-gal, and incubated overnight at 37°C [Gossen and Vijg,^1993^]. Concurrent titers on nonselective minimal agar were employed to enumerate total plaque-forming units (pfu). Mutant frequency was expressed as the ratio of the mutant plaques to total pfu. Preparation and exposure of primary hepatocytes from the Muta™Mouse was also conducted as described in Chen et al. [2010].

### Cytokinesis-Block Micronucleus Assay

The frequency of spontaneous and induced micronuclei (MN) was evaluated as described in Fenech [2005]. FE1 cells were seeded at a density of 2 × 10^5^ cells/plate and grown for 24 hr at 37°C in a 5% CO_2_ atmosphere. MN frequency was evaluated for each of the three replicate CSCs for each brand in duplicate for each dose. Cells were exposed in serum-free media for 1 hr to 90, 120, or 150 μg/mL CSC. DMSO was employed as the negative control (solvent blank) and mitomycin C was used as the positive control. The treatment was removed and cells were incubated for a further 24 hr in the presence of 3 μg/mL cytochalasin B (Sigma-Aldrich, Canada). Cells were removed from the growth surface, gently centrifuged, and resuspended in a 75 mM hypotonic KCl solution. Samples were fixed in 5:1 methanol:glacial acetic acid, and the cell suspensions dropped onto ice-cold slides, washed with fixative, and dried overnight. Slides were stained with Giemsa for microscopic examination. Two thousand binucleated cells were scored from each of two replicates for each CSC triplicate.

### Cell Exposures for DNA Microarrays

For microarray analysis, FE1 cells were propagated from one vial of cryo-preserved stock of passage #19. Experiments were performed on five replicates per condition (i.e., *n* = 5 per treatment group). Cells were exposed at 70% confluence in 150 mm plates to either 45 μg/mL or 90 μg/mL CSC or 1% v/v DMSO in 1:1 DMEM:F12 (without FBS). Cells were exposed for 6 hr and then either: (a) immediately harvested, or (b) washed with PBS and cultured for another 4 hr in fresh media. The two time points are referred to as 6 hr or 10 hr (i.e., 6 hr exposure with no recovery and 6 hr exposure with 4 hr recovery). Cells were harvested using TriZol® (Invitrogen Life Technologies, Canada) and stored at −80°C. Total RNA was isolated as described below.

### DNA Microarrays

#### RNA Extraction

Total RNA was extracted from control and treated FE-1 cells in a randomized fashion using Trizol Reagent (Invitrogen Life Technologies, Canada) according to the manufacturer's instructions. Subsequently, total RNA was further purified using the RNeasy Mini Kit (Qiagen, Canada) as directed by the manufacturer. The purified total RNA was resuspended in nuclease-free water. The concentration and quality of the RNA was assessed using a NanoDrop ND-1000 spectrophotometer and an Agilent 2100 Bioanalyzer. Sample purity was determined using the ratio of absorbance at 260 nm and 280 nm (*A*_260_/*A*_280_) and the RNA Integrity Number (RIN). All *A*_260_/*A*_280_ absorbance ratios were at least 2.0 and RINs ranged from 8.2 to 10 for all RNA samples.

#### DNA Microarray Hybridization

Total RNA samples from five independent replicates from each treatment group and time point were analyzed alongside matched solvent-exposed controls. A reference sample (Stratagene Universal mouse reference RNA) was hybridized to each microarray as an internal control, and for normalization. Double-stranded cDNA and cyanine labeled cRNA were generated according to the manufacturer's instructions (Agilent Linear Amplification Kits, Agilent Technologies, Canada). Experimental samples were labeled with cyanine 3-CTP, and reference RNA with cyanine 5-CTP (Perkin-Elmer Life Sciences, Canada). T7 RNA polymerase was used to transcribe cyanine-labeled cRNA targets, followed by purification with RNeasy Mini Kits (Qiagen, Canada). Labeled cRNA was hybridized to Agilent 22K mouse development microarrays (∼ 20,000 unique 60 mer oligonucleotides, Agilent Technologies, Canada) at 60°C overnight. Arrays were washed and scanned on a ScanArray Express (Perkin-Elmer Life Sciences, Canada) and data were acquired with ImaGene 5.5 (BioDiscovery Inc.).

### Normalization and Statistical Analysis

A reference design was used to analyze gene expression microarray data. Background fluorescence was measured using the (−)3xSLv1 probes; probes with median signal intensities less than the trimmed mean (trim = 5%) plus three trimmed standard deviations of the (−)3xSLv1 probes were flagged and called absent. Lowess normalization was carried out in R [R Development Core Team,^2004^]. Ratio intensity plots and heat maps for the raw and normalized data were constructed to identify outliers. Genes that were differentially expressed as a result of treatment were determined using two approaches. The first approach used the MAANOVA library in R [Wu et al.,^2003^]. The main effect in the model was treatment and the model was applied to the log_2_ of the absolute intensities. The Fs statistic was used to test for treatment effects [Cui et al.,^2005^]. The *P* values for all statistical tests were estimated by the permutation method using residual shuffling, followed by adjustment for multiple comparisons using the false discovery rate (FDR) approach [Benjamini and Hochberg,^1995^]. The fold change calculations were based on the least-square means. Significant genes were identified as having an adjusted *P* value <0.10 for any individual contrast.

The second microarray analysis, which was conducted independently of the first, used a different methodology. Fold changes were based on the arithmetic mean of exposed versus control within groups. This estimate of fold changes is different than that based on the least squared means described above, which used a linear model to estimate fold change. An ANOVA analysis was used to generate unadjusted *P* values. Fold changes and *P* values were used to explore the gene list, looking for trends in the data (e.g., direction and magnitude of fold changes across exposure conditions). Probe replicates (i.e., multiple identical probes for the same gene) were averaged following the statistical analysis.

Condition and gene trees were generated in GeneSpring 7.1 (Agilent Technologies) to explore the relationships among samples using various gene lists. Gene ontology (GO) enrichment was conducted on significant genes using the Database for Visualization, Annotation and Integrated Discovery (David; available at: http://david.abcc.ncifcrf.gov [Dennis et al.,^2003^; Huang da et al.,^2009^]). KEGG pathways were used to identify specific biological pathways associated with the differentially expressed genes using both the significant gene list and also a rank-based approach on all genes [Alvo et al.,^2010^].

### RT-qPCR Validation

RNA was extracted again for RT-qPCR and validation experiments were conducted in accordance with the MIQE (Minimum Information for Publication of Quantitative Real-Time PCR Experiments) guidelines [Bustin et al.,^2009^].

All primers were designed using Beacon Designer 7.60 (Premier Biosoft International), and primer sequences are available upon request. Temperature gradient PCR was conducted to determine the most appropriate *T*_m_ (°C) for each primer pair. Specific PCR amplification efficiencies, and correlation coefficients for each gene, were determined using five-point, 10-fold serial dilutions of pooled experimental cDNA to construct the calibration curves. PCR amplification efficiencies were all between 90 and 105%, and correlation coefficients were at least 0.985 for all primer pairs examined.

cDNA synthesis reactions were performed in triplicate. For each reaction, 5 μg of total RNA per sample was reverse transcribed using SuperScript® III Reverse Transcriptase and oligo(dT)_20_ primer (Invitrogen Life Technologies, Canada) following the manufacturer's instructions. Real-time PCR amplification reactions were performed in 96-well plates using a CFX96™ Real-Time PCR Detection System (Bio-Rad, Canada). RT-qPCR was performed in duplicate for each cDNA sample, using iQ™ SYBR® Green Supermix (Bio-Rad, Canada). A no-template control (NTC) was included for each gene on each plate to monitor for reagent contamination. The CFX_2StepAmp+Melt program (CFX Manager Software, Bio-Rad, Canada) was used to validate the expression patterns of the following 15 genes: *Cdc20*, *Cdkn1*, *Cyp1b1*, *Egr1*, *Egr2*, *Fosl1*, *Il6*, *Il10*, *Myc*, *Nqo1*, *Nr4a1*, *Plk1*, *Socs1*, *Socs2* and *Tgfb2*, and the CFX_3StepAmp+Melt program was used to validate the expression patterns of the remaining six genes: *Cyp1a1*, *Ddit3*, *Gadd45a*, *Gadd45b*, *Prc1*, and *Sesn1*. Specific conditions are available upon request. PCR efficiency was examined using the standard curve for each gene. The threshold cycle (*C*_t_) values for duplicate reactions were averaged.

Hypoxanthine guanine phosphoribosyl transferase 1 (*Hprt1*) was selected for use as a reference gene as its expression was shown to be stable across all experimental conditions by gene expression microarray analysis and by RT-qPCR analysis using a subset of representative samples. Gene expression levels were normalized to the *Hprt1* gene.

### Statistical Data Analyses

Ordinary, least-squares linear regression of the initial linear portion of the concentration–response functions was used to determine Salmonella mutagenic potency values in revertants per μg TPM. For comparison with other studies the data presented in Table II, which provide the tar and nicotine content (mg/cig) for each brand investigated, can be used to convert mutagenic potency values from revertants/μg TPM to revertants/mg tar or revertants/mg nicotine. Data from Table I can be used to convert potency values to revertants per cigarette. Two sample t-tests assuming unequal variances, with the appropriate Bonferroni correction, were used to contrast strain- and brand-specific mutagenic potency values. *LacZ* mutation and micronucleus frequency data were analyzed by Poisson regression using SAS version 9.2, and the data were fit to the model log(*E*(*Y*_*i*_)) = log *t*_*i*_+ β**x**_*i*_, where *E*(*Y*_*i*_) is the expected value for the *i*th observation, β is the vector of regressions coefficients, **x**_*i*_ is a vector of covariates for the *i*th observation, and *t*_*i*_ is the offset variable used to account for differences in observation count period (i.e., pfu or binucleate cells scored). The offset (i.e., natural log of pfu or binucleate count) was given a constant coefficient of 1.0 for each observation and log-linear relationships between mutant count or micronucleus count and test mutagen concentration were specified by a natural log link function. Type 1, or sequential analysis, was employed to examine the statistical significance of the chemical treatment, and custom contrasts were employed to evaluate the statistical significance of responses at selected concentrations. Custom contrasts were accomplished by specifying an L matrix, and computing statistics for pair wise comparisons based on the asymptotic χ^2^ distribution of the likelihood ratio. The results of post hoc pair-wise comparisons were interpreted using the Holm-Bonferroni correction for multiple comparisons.

**TABLE II:** Levels of Selected Analytes, Including Several Carcinogens, in Mainstream Emissions^a^ from the Cigarette Brands Examined

Analyte^b^	Brand 1	Brand 3	Brand 5	Carcinogenicity^c^
Tar (mg/cig)	15.6	12.9	12.4	NA
Nicotine (mg/cig)	1.3	1.1	1.1	NA
CO (mg/cig)	14.0	13.6	12.7	NA
Benzo[a]pyrene (ng/cig)	9	8	10	1
4-Aminobiphenyl (ng/cig)	2	2	2	1
3-Aminobiphenyl (ng/cig)	3	3	3	NC
2-Aminonaphthalene (ng/cig)	11	11	11	1
Pyridine (μg/cig)	19	16	11	3
NNN (ng/cig)	37	178	25	1
NNK (ng/cig)	75	63	52	1
Cadmium (ng/cig)	90	47	90	1
Lead (ng/cig)	NQ	19	NQ	2B
Formaldehyde (μg/cig)	82	54	44	2A
Acetaldehyde (μg/cig)	698	680	587	2B
1,3-butadiene (μg/cig)	52	44	48	2A
Isoprene (μg/cig)	276	376	301	2B
Acrylonitrile (μg/cig)	11	12	10	2B
Benzene (μg/cig)	49	43	49	1
Styrene (μg/cig)	14	10	10	2B

a International Organization for Standardization (ISO) standard 3308. Data from Controlled Substances and Tobacco Directorate, Health Canada, 2004.

b NNN, *N*2-nitrosonornicotine; NNK, 4-(methylnitrosamino)-1-(3-pyridyl)-1-butanone; NQ, not quantifiable (above the limit of detection but below the limit of quantitation).

c According to IARC monographs 29, 32, 58, 71, 82, 84, 87, 88, 89, 92, and supplements. NA indicates not applicable. NC indicates not classified by IARC. 1 indicates carcinogenic to humans, 2A indicates probably carcinogenic to humans, 2B indicates possibly carcinogenic to humans.

The mathematical model used to calculate gene expression was the efficiency corrected calculation model [Pfaffl,^2006^]. Gene expression validation of microarray data was conducted by calculating the relative gene expression ratios for all 21 genes using REST08 (Relative Expression Software Tool 2008, Corbett Research). This software allows for the comparison of relative quantification between treatment groups, and calculates the significance of the differences using a Pair Wise Fixed Reallocation Randomization Test© [Pfaffl et al.,^2002^].

## RESULTS

The numbers of cigarettes smoked to obtain the CSC samples, and the TPM yield, both total and per cigarette, are summarized in Table I. In addition, Table I provides information on tobacco types and the manufacturer's brand descriptions (i.e., light, full-flavor, blonde). Table II summarizes the concentrations of selected analytes in mainstream emissions from each of the products examined. Since tar levels in Canadian benchmark cigarettes range from 0.7 to 15 mg/cigarette [Health Canada,^2011^], the tar values for the brands examined here indicate that all three would be considered high tar brands. However, Brand 1 is a high-tar Virginia flue-cured brand marketed as full-flavor (i.e., not ventilated), Brand 3 is a high-tar blonde brand containing mixed tobacco, and Brand 5 is a high-tar ventilated Virginia flue-cured brand marketed as “light.” The highest TPM yield and tar content was noted for Brand 1, followed by Brand 3 and Brand 5, although there was little difference between the latter two. In addition, Table II provides the IARC carcinogenicity classification for select analytes. The concentrations of chemical analytes in the different CSCs are quite similar, however, some notable differences include: (a) *N*-nitrosonornicotine (NNN), lead, and isoprene were highest and cadmium lowest in Brand 3, (b) formaldehyde and 4-(*N*-methylnitrosamino)-l-(3-pyridyl)-l-butanone (NNK) were highest in Brand 1, and (c) Brand 5 tended to have similar or lower levels of most analytes relative to the other brands.

### Salmonella Mutagenicity

The Salmonella mutagenicity analyses showed a mean spontaneous reversion frequency of 37.3 ± 1.7, 42.9 ± 2.7, and 30.5 ± 1.0 rev/plate ± SEM for TA98, YG1041, and YG5161, respectively, in the presence of metabolic activation. Mean response to the positive control (2AA) was 525.8 ± 51.1, 387.6 ± 63.9, and 973.1 ± 130.3 rev/plate for TA98 (0.5 μg/plate), YG1041 (0.1 μg/plate), and YG5161 (0.5 μg/plate), respectively.

**Fig. 1:**
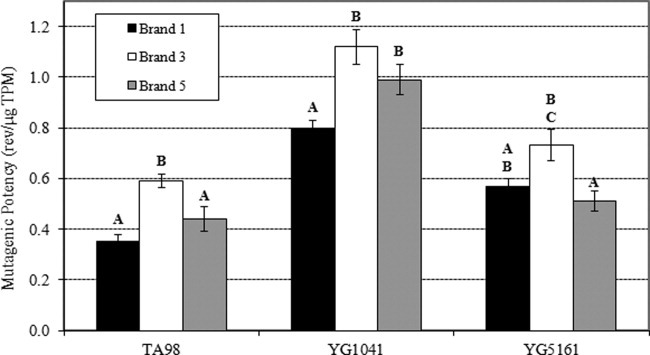
Salmonella mutagenicity of CSC samples representing three cigarette brands. Values shown are mean mutagenic potency values, in revertants per μg TPM ± standard error, for TA98, YG1041, and YG5161 with S9 activation. Bars accompanied by the same letter are not significantly different at *P* < 0.005. Employing the appropriate Bonferroni correction the critical *P* value is 0.0167.

All CSC samples were mutagenic in the standard frameshift test strain TA98, as well as in YG1041, which has enhanced sensitivity to nitroarenes and aromatic amines, and YG5161, which has enhanced sensitivity to unsubstituted PAHs (Fig. 1). TA98 potency values were always lowest (0.35–0.59 rev/μg TPM), followed by YG5161 (0.51–0.73 rev/μg TPM), and YG1041 (0.80–1.12 rev/μg TPM). For all brands investigated, mutagenic potency values obtained for YG1041 were significantly greater than those for both TA98 and YG5161 (*P* < 0.0001). An increase in mutagenic potency on YG1041, relative to TA98, indicates that aromatic amines are an important determinant of CSC mutagenic activity. In addition, an increase in mutagenic potency on YG5161, relative to TA98, indicates that unsubstituted PAHs are also determinants of CSC mutagenic activity. With respect to the brands, Brand 3 consistently yielded the highest mutagenic potency value. The results of the statistical comparisons across brands for the three Salmonella strains are shown in Figure 1. The results show that Brand 3 is significantly more potent than Brands 1 and 5 on TA98 (*P* < 0.0001 and *P* < 0.01), significantly more potent than Brand 1 on YG1041 (*P* < 0.0001), and significantly more potent than Brand 5 on YG5161 (*P* < 0.004). In addition, Brand 5 was significantly more potent than Brand 1 on YG1041 (*P* < 0.006). In summary, mutagenic potency is not markedly different across the brands, although Brand 3 elicited the highest response.

### FE1 Cytotoxicity

**Fig. 2:**
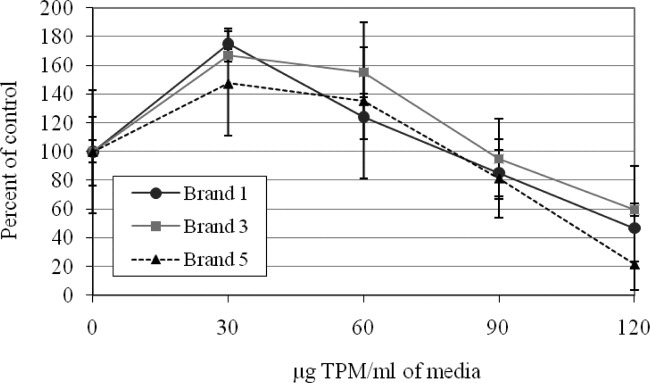
Cytotoxicity of CSC samples representing three cigarette brands. The response variable indicates clonogenic survival relative to the solvent control (i.e., 100%). Error bars represent the standard error of the mean.

The results of the cytotoxicity analyses are illustrated in Figure 2. The low doses of CSC (i.e., at concentrations less than 90 μg TPM/mL) appear to be stimulatory (i.e., relative clonogenic survival >100%). More specifically, at the lower concentration used for toxicogenomic analyses (i.e., 45 μg TPM/mL), the CSC exposures elicited a 40 to 60% increase in clonogenic survival relative to control. Interestingly, at 30 μg TPM/mL, the peak of the inverted U-shaped concentration–response, the CSC exposures elicited 75, 67, and 48% increases in clonogenic survival values, relative to control, for Brands 1, 3, and 5, respectively. This type of biphasic concentration–response, which shows an increase in survival at low concentrations that is generally less than twofold greater than the control, is consistent with the compensatory response phenomenon described by Calabrese and Baldwin [2002].

The results for Brand 1 indicate no reduction in survival at concentrations less than approximately 78 μg TPM/mL, and Brands 3 and 5 did not elicit reduced survival at concentrations less than approximately 88 and 80 μg TPM/mL, respectively. At 90 μg TPM/mL, all brands showed slight cytotoxicity and yielded clonogenic survival values of 85, 95, and 82% of controls for Brands 1, 3, and 5, respectively. Above 90 μg TPM/mL, all brands showed substantial cytotoxicity, and yielded survival values that rapidly drop to below 60% of control. However, statistical analyses showed a significant drop in clonogenic survival at the highest concentration, relative to the control, only for Brands 1 and 5 (*P* < 0.05, one-sided t-test). Statistical analyses failed to reveal any significant differences in cytotoxicity between the three brands.

### FE1 LacZ mutagenicity

The *LacZ* transgene mutagenicity assay in FE1 cells showed a mean spontaneous mutant frequency (±standard error) of 34.3 ± 3.8 and 22.8 ± 0.7 × 10^−5^, without and with exogenous S9 activation, respectively. The mean response for the positive controls was 693.5 ± 36.4 and 88.3 ± 6.4 × 10^−5^ for 0.4 μM BaP and 2 μM PhIP, respectively.

The mutagenic activity of the CSC samples on the *LacZ* transgene mutagenicity assay was assessed across a range of concentrations (10–200 μg/mL) using three approaches: (1) in the absence of any external metabolic activation (i.e., S9), (2) in the presence of exogenous metabolic activation, using an activation mixture containing 0.5, 1, 2, or 4% (v/v) rat liver S9, and (3) preincubation of CSC samples with a rat liver metabolic activation mixture for 15, 30, or 60 min at 37°C before exposure of FE1 cells. Regardless of the approach, the results failed to detect any significant increase in *LacZ* mutant frequency in response to CSC exposure for any brand. Cytotoxicity studies conducted previously in our lab with other CSC showed that the use of higher concentrations (160 and 200 μg TPM/mL) resulted in little to no surviving cells. Therefore, despite the use of sufficiently high exposure concentrations, the CSCs examined failed to induce *LacZ* mutations in FE1 cells.

### Micronucleus Frequency in FE1 Cells

**Fig. 3:**
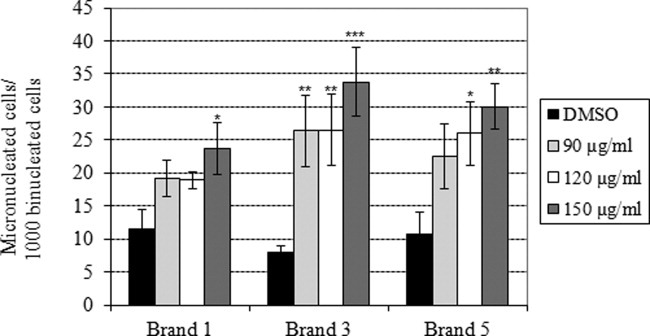
Clastogenicity of CSC samples representing three cigarette brands. Values shown are mean numbers of micronucleated cells per 1,000 scored binucleated cells. Data were analyzed using Poisson regression, and the symbols show the results of one-way post hoc contrasts with concurrent control, corrected using the Holm-Bonferroni method. *Significant increase at *P* < 0.05, **significant increase at *P* < 0.025, and ***significant increase at *P* < 0.01.

The cytokinesis-block micronucleus assay in FE1 cells showed a mean spontaneous frequency of micronucleated cells of 10.1 ± 1.1 cells per 1,000 scored binucleates. Exposure of FE1 cells to CSC resulted in an increase in overall MN frequency, suggesting that all brands were capable of inducing cytogenetic damage (Fig. 3). The most potent activity was observed for Brand 3, followed by Brand 5 and Brand 1. Brand 1 elicited the weakest response, with only the highest tested concentration yielding a response significantly greater than the solvent control.

### DNA Microarrays

#### Gene Expression Microarray MAANOVA Analysis

For gene expression analysis, FE1 cells were exposed to 45 or 90 μg TPM/mL for 6 hr. The 90 μg TPM/mL was selected because it is the lowest concentration that elicits a modest increase in cytogenetic damage (significantly elevated for Brand 3 only) without any significant increase in cytotoxicity (Figs. 2 and 3). In stark contrast, the 45 μg/mL concentration reflects a concentration that elicited a 40 to 60% increase in clonogenic survival relative to control. Selection of this concentration permitted gene expression analysis of a concentration that does not induce cytotoxicity or chromosome damage, and moreover, permits a preliminary investigation of the mechanism underlying the observed biphasic, compensatory response (Fig. 2). After 6 hr of exposure, cells were either collected into Trizol (6-hr time point), or washed and cultured in fresh serum for an additional 4 hr (10-hr time point). Analysis of MA plots and cluster analysis of the normalized signal intensities for all probes revealed a few outliers (i.e., unacceptable arrays, data not shown) that were eliminated from the analyses. MAANOVA was applied to at least four replicates in each treatment group to identify differentially expressed genes. Genes were considered significant if they yielded an FDR-adjusted *P* value <0.10, and a fold change greater than 1.5.

A total of 395 unique probe identifiers were significantly differentially expressed (i.e., up- or down-regulated in exposed samples compared with their matched controls at either one or both of the time points examined). Of these, 328 genes were deemed “present” (full list in Supporting Information Table S1). There were 47 genes that were disregulated at the low concentration and 319 at the high concentration; 38 genes were in common between the two concentrations.

Overall, gene expression was most altered at the 10-hr time point for each brand and concentration, relative to the 6-hr time point. In total, there were 115 genes identified as differentially expressed in at least one condition at 6 hr (54 down- and 61 up-regulated), and 254 at the 10-hr time point (172 down- and 82 up-regulated). The overall number of differentially expressed genes was relatively similar among the brands (Table III; Supporting Information Table S1). Venn diagrams showing the overlap of significantly differentially expressed genes among brands, within time points and concentrations, are shown in Supporting Information Figure S1A–S1D, and demonstrate a substantial overlap across the brands. The largest effect on gene expression was found for 90 μg/mL Brand 1 at the 10-hr time point, and the majority of the genes were down-regulated in this condition (171 of 237). However, in general, relatively similar numbers of genes were differentially expressed within a concentration and time point across the brands.

**TABLE III:** Total Number of Genes Up- or Down-Regulated (FDR *P* < 0.1 and Fold Change > 1.5) for each Brand Within Time Point and Concentration

	6 Hr				10 Hr			
		
	45 μg/mL		90 μg/mL		45 μg/mL		90 μg/mL	
				
	Up-regulated	Down-regulated	Up-regulated	Down-regulated	Up-regulated	Down-regulated	Up-regulated	Down-regulated
Brand 1	5	3	22	25	24	5	68	171
Brand 3	2	0	54	30	13	11	53	47
Brand 5	4	12	37	42	8	7	82	103

**Fig. 4:**
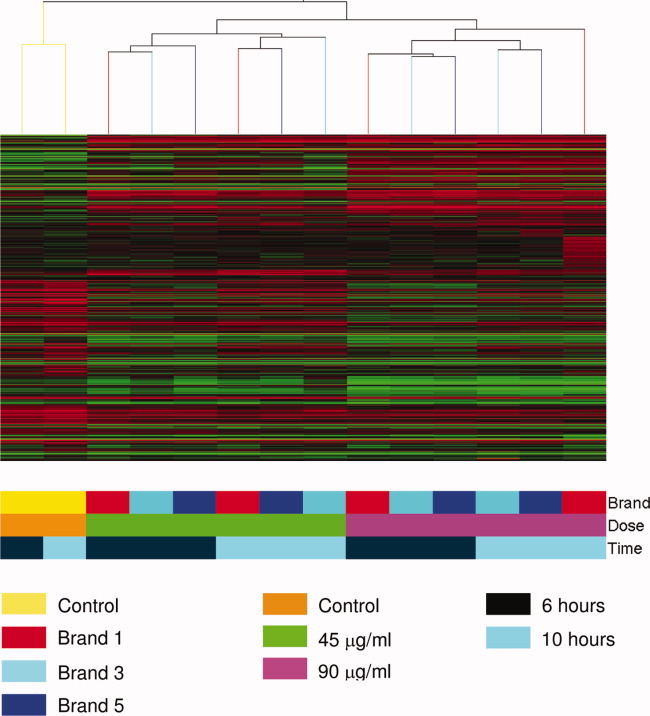
Heat map of the mean expression of the 328 differentially expressed probes across the concentrations, time points and brands. Red represents high expression relative to the reference sample, and green represents low expression relative to reference. The cluster analysis reveals that the expression profiles cluster first by concentration, followed by treatment.

A condition tree (cluster analysis) was used to examine the influence of brand, concentration, and time on expression profiles. The analysis revealed that samples clustered first by concentration, followed by time, with brand having the smallest effect on the expression profiles. Visual inspection of the cluster analysis suggests that expression profiles were similar across the brands within a concentration and time point (Fig. 4).

The top 10 most differentially regulated genes (i.e., largest fold changes) were: TCDD-inducible poly(ADP-ribose) polymerase (*Tiparp*; 12-fold up-regulated), B-cell translocation gene 2, antiproliferative (*Btg2*; 10 fold up-regulated), tetraspanin 33 (*Tspan33*; 10-fold up-regulated), DNA-damage inducible transcript 3 (*Ddit3*; 10-fold up-regulated), arrest in domain containing 3 (*Arrdc3*; ninefold up-regulated), serine (or cysteine) proteinase inhibitor, clade E, member 1 (*Serpine1*; ninefold up-regulated), insulin-like growth factor 1 (*Igf1*; eightfold up-regulated), hemeoxygenase (decycling) 1 (*Hmox1*; eightfold up-regulated), similar to Crb2 protein (*Crb2*; eightfold down-regulated), cytochrome P450, family 1, subfamily a, polypeptide 1 (*Cyp1a1*; sevenfold up-regulated), and polo-like kinase 1 (Drosophila) (*Plk1*; sevenfold down-regulated). With the exception of *Plk1*, all these genes were differentially regulated for all time points, concentrations, and brands, although not always attaining FDR-adjusted statistical significance.

GO and pathway analysis of all the differentially expressed genes revealed significant pertubations associated with cell cycle, p53 signaling, apoptosis/programmed cell death, and steroid/cholesterol biosynthesis (Supporting Information Table S2A). Functional annotation clustering was conducted in order to minimize redundancy among the GO terms. This analysis revealed 23 clusters with enrichment scores greater than 1.5 (Supporting Information Table S3). The top cluster was associated with cell cycle and mitosis (enrichment score 9.09; see also clusters 8, 12 and 16, 18 for cell cycle and mitosis, respectively), followed by cholesterol/steroid metabolic processes (enrichment score 3.25). Other clusters relevant to the induced DNA damage, cytotoxicity, and cell cycle-related effects included chromosomal condensation/segregation (clusters 5, 11, 13, 16, 17, 21; enrichment score 2.45), and regulation of apoptosis and cell death (both negative and positive regulation; cluster 6, 9; enrichment score 2.43). Thus, the analysis revealed the principal pathways of cell cycle, mitosis, and chromosome segregation, as well as apoptosis and cell death.

To investigate early versus downstream effects, and to differentiate between processes that were induced versus repressed, GO analyses were applied to individual time points on up-regulated versus down-regulated genes. At the 6 hr time point, GO analysis identified 14 significant biological functions associated with down-regulated genes (Supporting Information Table S2A). Twelve of these 14 were associated with cell cycle, replication, and division including, for example, the GO terms cell cycle, mitosis, M phase, chromosome segregation, and spindle. Analysis of up-regulated genes at 6 hr revealed three Kegg pathways with Benjamini-Hochberg-adjusted *P* values <0.05, and included p53 signaling, pathways in cancer, and bladder cancer (Supporting Information Table S2B). Twelve GO terms were enriched among up-regulated genes and included cell death, cellular response to stress, and transcription factor activity.

At 10 hr (i.e., 4 hr postexposure), down-regulated genes were enriched for 13 GO terms, the majority of which were again associated with cell cycle (Supporting Information Table S2C), while the rest were related to steroid metabolism. Down-regulated genes were enriched for several Kegg pathways including: cell cycle, p53 signaling, oocyte meiosis, and ubiquitin-mediated proteolysis. DAVID analysis of up-regulated genes at 10 hr revealed four GO terms, including terms associated with cell death/apoptosis, and enrichment for the p53 signaling response Kegg pathway (Supporting Information Table S2D).

Examination of the genes that responded at the low concentration did not reveal any significantly enriched functional annotation terms after adjustment for multiple comparisons. However, pathways with nonadjusted *P* < 0.05 included oxidative stress response, regulation of apoptosis and cell death, cell cycle, and metabolism of xenobiotics by cytochrome p450 (data not shown). Thus, although fewer genes were differentially regulated at the low concentration, the molecular pathways and functions affected appeared to be consistent with response to the high concentration.

### Genes Associated With Compensatory Mechanisms

Because we noted increased cell survival at the low concentration, we examined genes that were more affected at the low concentration relative to the high concentration to provide insight into the compensatory mechanism. Several genes showed large increases in expression between the control and low concentration, with substantially smaller increases, or decreases, relative to control, at the high concentration. For example, genes involved in xenobiotic metabolism and antioxidant defense, such as C*yp1a1*, *Cyp1b1*, and *Nqo1*, show two to fivefold declines in expression from low to high concentration (Table IV). Several genes involved in cell cycle regulation, including *Cdc20*, *Plk1*, and *Prc1*, also showed large (i.e., 5- to 14-fold) declines in expression between the low and high concentrations. These latter genes are all known to be positive regulators of replication, cell division, and cell proliferation [Mollinari et al.,^2002^; Jang et al.,^2007^]. In contrast, several genes involved in p38/JNK-dependent pathways showed continual increases in expression from control to high concentration [Hildesheim and Fornace,^2002^]. These include antiproliferative, proapoptotic, and/or DNA damage inducible genes such as *Gadd45a*, *Gadd45b*, *Ddit3*, *Junb*, *Cdkn1a* (*p21*), *Atf3*, and *Fosl1*. Thus, the trends in gene expression profiles reflects a low concentration compensatory response involving metabolism, antioxidant defense, and growth stimulation, followed by a high concentration cytotoxic response involving p38/JNK-dependent DNA repair, cell cycle arrest, and apoptosis.

**TABLE IV:** Fold Change Values for Microarray (Supporting Information Tables S1 and S5) and RT-PCR Data Presented for each Time, Concentration, and Brand for 22 Genes

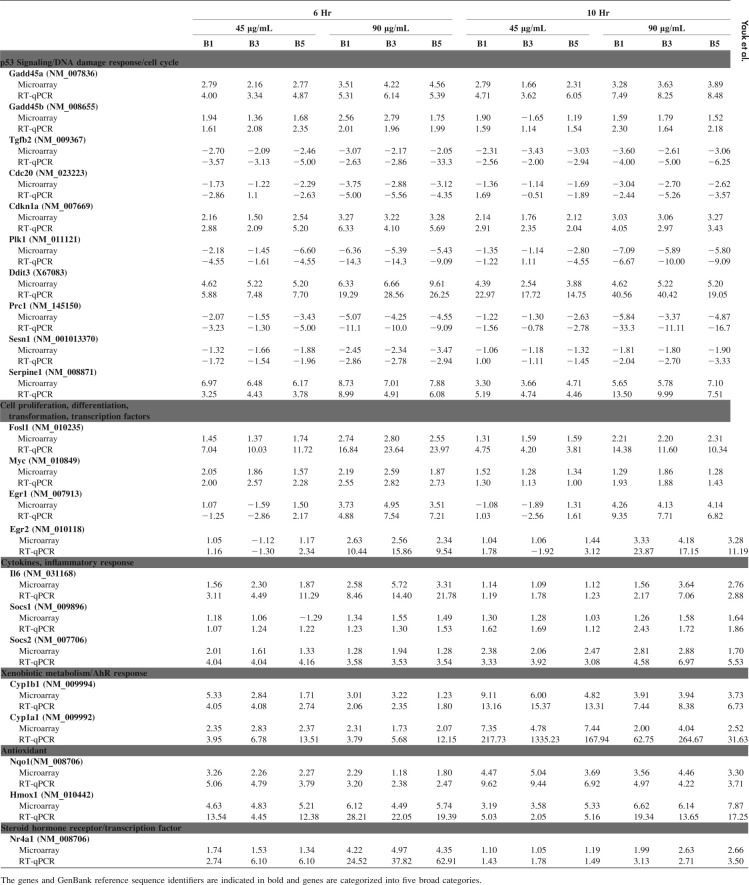

### Gene Expression Microarray Analysis by Arithmetic Mean of Fold Changes

Previous work by various authors has suggested that analysis of microarray data by fold change provides the most reproducible data [Guo et al.,^2006^]. As such, we also applied a more liberal approach to see whether we could identify brand-specific effects that were not identified using the stringent MAANOVA analysis. Arithmetic means of fold changes, with unadjusted *P* values calculated using t-tests, and the data were filtered to examine similarities and differences across brands. We identified approximately 1,600 probes that showed similar changes in direction of expression consistently across all brands and time points relative to time-matched controls (Supporting Information Table S4A). These were eliminated from our search for brand-specific effects. From the remaining probes, genes with a fold change of at least 1.5 in any one contrast were retained for analysis of brand-specific effects (Supporting Information Table S4B). These genes were examined in detail to determine whether there was any strong evidence for brand-specific responses (e.g., up- or down-regulated in one brand only, or differentially regulated across all but one brand within time points, etc.). This analysis did not generate any convincing examples of differences in expression profiles among the CSC examined.

### RT-qPCR Validation of Microarray Results

A large subset of differentially expressed genes was selected for RT-qPCR validation that spanned some of the major pathways affected by the brands. In total, 22 genes were selected from both the MAANOVA (Supporting Information Table S1) and the fold change rank (Supporting Information Tables S4A and S4B). These genes were selected based on involvement in the following processes: (a) p53 signaling/DNA damage response/cell cycle, (b) cell proliferation, differentiation, transformation, transcription factors, (c) cytokines, inflammatory response, (d) xenobiotic metabolism/AhR response, (e) antioxidant activity, and (f) steroid hormone receptor/transcription factor. RT-qPCR was conducted on these genes to confirm the expression changes measured by the DNA microarrays, in addition to searching for any clear differences among the treatment groups for these specific genes. The results of this analysis are presented in Table IV and reveal a remarkable consistency in response across brands for each time and concentration. Although fold changes tended to be larger for RT-qPCR, the data demonstrate a high degree of concordance between the DNA microarray and RT-qPCR findings, and no clear brand-specific differences.

## DISCUSSION

The present study constitutes a comprehensive, comparative study of CSCs from three tobacco products available in Canada. The findings failed to reveal any striking differences between the brands for any of the endpoints examined, providing further support for the notion that tobacco products marketed as “light” provide no clear evidence of hazard reduction.

### Cytotoxicity and Mutagenicity of the CSCs

The cytotoxicity and mutagenicity results presented here support previous observations that CSC is mutagenic and cytotoxic in both bacterial and mammalian cells [DeMarini,^1983^,^2004^; Foy et al.,^2004^; DeMarini et al.,^2008^]. Previous studies have shown that the Salmonella mutagenic potency of CSCs is approximately 0.5 to 2.5 revertants/μg TPM [Steele et al.,^1995^; Chepiga et al.,^2000^; Rickert et al.,^2007^; DeMarini et al.,^2008^]. This is consistent with our results (Fig. 1) showing a range of potencies from approximately 0.4 to 1.1 revertants/μg TPM. The results presented here indicate that the response was highest on the Salmonella strain YG1041, highlighting the importance of aromatic amines in contributing to the mutagenicity of CSCs derived from all three brands. Although all brands were mutagenic, a significantly higher response was observed for Brand 3. However, despite slight differences in mutagenicity, the range of potencies was small. Analysis of cytotoxicity in cultured FE1 pulmonary epithelial cells demonstrated that the CSCs were only slightly toxic at concentrations less than 90 μg/mL. Moreover, as noted earlier, clonogenic survival at lower concentrations was observed to be greater than the control. This latter result is similar to that presented by Foy et al. [2004]. Their assessment of neutral red uptake by CHO-WBL cells exposed for 24 hr to CSCs from six different tobacco products indicate that at 10 μg/mL, cytotoxicity, expressed as percentage of control, reached as high as 117%. The difference between this value and the low concentration compensatory responses observed in this study (i.e., up to 178% of control) is likely due to the differential sensitivities of the assay employed. Cytotoxicity endpoints such as neutral red uptake, Trypan Blue exclusion, or MTT reduction have been highlighted for their lack of sensitivity relative to endpoints that assess replication and clonal survival [Rossman,^2009^].

Despite repeated attempts across a broad range of concentrations, with and without exogenous metabolic activation, we were unable to measure a significant increase in mutant frequency at the *LacZ* locus in exposed FE1 cells. At higher concentrations, the CSCs were highly cytotoxic, and we were unable to retrieve adequate amounts of DNA for mutation analysis. These results are consistent with our previous work using primary hepatocytes derived from the Muta™Mouse for the Brand 5 CSC at 80, 120, and 160 μg/mL (reported elsewhere) [Chen et al.,^2010^]. *LacZ* mutant frequency in exposed primary hepatocytes showed a small but statistically significant increase (1.6-fold, *P* < 0.005) for the low concentration only. At higher concentrations, the primary hepatocytes showed reduced cell survival and a low yield of extractable DNA. Guo et al. [2011] also observed high cytotoxicity of CSCs in mammalian cells employed for mutagenicity analyses. In an analysis of 11 CSCs, these authors found that all brands exhibited relatively similar mutagenic potencies for the *Tk* mutation assay in mouse lymphoma cells (L5178Y), suggesting that CSCs can induce mutations in mammalian cells exposed in vitro. However, they also noted that the CSCs are active across a narrow concentration range due to their high cytotoxicity. Other studies that examined induction of mutations in mammalian cells (e.g., the endogenous *hprt* locus) exposed to CSCs have yielded mixed results. Krause et al. [1999] found a significant increase in mutations in MCL-5 cells, which carries two recombinant plasmids expressing xenobiotic metabolizing enzymes, following exposure to CSC. However, Doolittle et al. [1990] failed to detect induced *hprt* mutations in CHO cells exposed to CSCs both with and without metabolic activation. Jongen et al. [1985] noted significant induction of *hprt* mutations in V79 cells exposed to CSC, but only in the presence of exogenous metabolic activation. Thus, the data presented here for FE1 cells, combined with the aforementioned observations of Chen et al. for primary hepatocytes from Muta™Mouse, and the mixed results presented in the literature, indicates that CSC can induce gene mutations in mammalian cells. However, successful detection of mutation induction occurs over a narrow concentration range in systems that have the appropriate metabolic capacity. To date, the exact biochemical nature of this metabolic requirement has not been well defined.

Our results indicate that MN formation can be induced in the FE1 cell line following exposure to CSCs. The clastogenic potencies were 0.0076, 0.017, and 0.013 micronucleated cells/μg CSC for Brands 1, 3, and 5, respectively. These results are consistent with published in vitro exposures to whole cigarette smoke [Massey et al.,^1998^] and CSC [Channarayappa et al.,^1992^; Gu et al.,^1992^; DeMarini et al.,^2008^] that consistently show induction of chromosome damage in the form of MN. DeMarini et al. [2008] examined CSCs from 10 cigarette brands and noted that all samples induced MN in mouse lymphoma cells with a less than a 3-fold range in potency (expressed as MN per μg CSC). The values presented here are similar to those published by DeMarini et al., and, with a 2.2-fold range in potency across the brands, supports the notion that brand-specific clastogenic potency values do not show substantial variability. Lou et al. [2010] recently assessed cytotoxicity, apoptosis, DNA strand breaks, and MN formation in cultured human β-lymphoblastoid cells exposed to CSCs from 12 brands. Their results revealed induction of cytotoxicity and apoptosis for all 12 CSCs. Eleven caused strand breaks in a concentration-dependent fashion as measured by the comet assay, and nine brands were positive in the MN assay. Moreover, these authors noted a high degree of correlation among the potency rankings for the assays. In addition, the range of potency values was consistent across the assays, except for the comet assay, which demonstrated higher variability.

### Gene Expression Profiling

Global gene expression signatures were investigated to examine whether brand-specific signatures could be identified using a toxicogenomics approach, to establish baseline gene expression profiles for the brands examined, and to provide insight into the mechanisms underlying the responses to CSC. The expression profiles revealed alterations in genes associated with cell cycle/DNA replication (primarily down-regulated at the higher concentration). In keeping with the known mechanism of action of various chemical toxicants in CSC, including PAHs and nitrosamines, we noted up-regulation of genes involved in xenobiotic metabolism, oxidative stress response and DNA damage response. Although the response was greatest at the high concentration (i.e., more genes and larger changes), the responsive biological processes were similar between the high and low concentrations. Nevertheless, it is important to note that concentration-response trends for several genes were observed, and this pattern, as well as the aforementioned overall gene expression pattern, is consistent with the cytotoxicity and mutagenicity results. The concentration-related changes in gene expression revealed that a subset of genes, including some involved in xenobiotic metabolism and antioxidant defense, replication, cell division, and cell proliferation, show marked decreases in expression between the low and high concentrations. Detailed examination of these changes indicates that the observed trends are consistent with the compensatory cytotoxicity response observed at the low concentration (i.e., increased clonal survival at the low concentration relative to the unexposed control). In contrast to previous publications [Lu et al.,^2007^; Pickett et al.,^2010^], gene expression profiles were highly similar across the three brands. Although certain brands yielded slightly more differentially expressed genes, the trend, in both direction and magnitude, of the fold changes were similar across all brands. Thus, CSCs from all three brands, including the brand marketed as “light” (i.e., Brand 5), exhibited highly similar gene expression profiles.

To our knowledge, two previous studies have used global transcriptomic analyses to attempt to discern brand-specific toxicogenomic profiles and identify candidate biomarker genes. These studies produced lists of genes that were hypothesized to potentially be useful in assessing exposures to certain brands or types of cigarettes (e.g., varying tar-content). Pickett et al. [2010] explored gene expression changes in cultured primary human bronchial epithelial cells (collected from one individual) exposed to CSCs from 10 brands of cigarettes. Cells were exposed to CSCs for 18 hr, and exposures were based on nicotine concentration (4 μg/mL). In contrast, the exposure design in our experiment was based on CSC concentration, and nicotine concentrations were approximately 4.3, 5.9, and 7.0 μg/mL for the high concentrations of Brand 1, Brand 3, and Brand 5, respectively (1.6-fold variation in nicotine content across our study). Thus, the nicotine exposure concentrations used in Pickett et al. were similar to our study. Pickett et al. identified 21 genes that appeared to be differentially regulated across most brands in the primary human bronchial cells. These genes included *Nqo1*, *Cyp1a1*, *Cyp1b1*, *Akr1c1/c2* (we found *Akr1c18*), *Angptl4*, *Fbxo32* (we found *Fbxo5*), *Gdf2* (we found *Gdf9* and *Gdf15*), and *Cxcl14*, which were also identified as differentially expressed in the current study. Thus, approximately 40% of the genes identified in the Pickett et al. study are consistent with our work, despite being conducted in different cells from a different species exposed for 18 hr, rather than 6 hr. Pickett et al. identified genes that they proposed are unique to the various brands by using a twofold threshold and a Venn diagram approach. However, the analysis was limited by a lack of biological and/or technical replicates (i.e., duplicates only), a single concentration, and a single time point. Therefore, although the authors found some genes that appeared to show a unique response to individual brands, these findings should be interpreted with caution as additional concentrations and time points are necessary to confirm the results.

Lu et al. [2007] compared cytotoxicity (neutral red assay) and global gene expression in mouse Balb/3T3 fibroblast cell cultures exposed to CSCs using an in vitro whole smoke exposure system. Cells were exposed for 1 hr to three commercial cigarette brands (one full-flavor, one low tar, and one ultra-low tar), and one type of reference cigarette, at wet total particulate matter levels that gave similar cytotoxicities (10–20%) and nicotine concentrations across the cigarette types. Samples were analyzed 5 hr after a 1 hr exposure. The authors found that cytotoxicity decreased with tar content, and identified a total of 598, 176, and 234 differentially expressed genes for the full-flavor, low tar, and ultra-low tar cigarettes, respectively. The pathways and processes perturbed in these cells are similar to those identified here. Inflammatory and glutathione reduction processes were up-regulated and cell proliferation/replication pathways were down-regulated. The authors found that the latter two pathways exhibited what appeared to be a brand-specific response, with high tar content cigarettes having the greatest effect on cell cycle, and using class predictions and a set of 100 selected “predictor” genes, they suggested that a transcriptomic analysis could be used to correctly classify exposed samples into cigarette groups. However, this work analyzed only three brands, and it would be necessary to verify the utility of the identified predictor genes.

The results of the two studies described above are consistent with the present study in terms of the pathways affected by CSCs. First, there is a large overlap in the genes, gene families, and biological processes observed in our study in comparison with these studies. In our study, the largest number of differentially expressed genes was found for Brand 1 (237 genes), a full-flavor cigarette containing Virginia flue-cured tobacco that has the highest tar and nicotine content of the three brands examined. This is consistent with Lu et al. [2007]. Indeed, this brand had approximately two times as many genes meeting our statistical threshold, suggesting that it induced a more substantial overall cellular effect. Moreover, in keeping with Lu et al., the majority of the genes perturbed by Brand 1 CSC were involved in cell cycle control. Although Brand 5 and Brand 3 (which are different tobacco blends) each have similar levels of tar (12.4 and 12.9 mg/cig, respectively) and nicotine (both have 1.1 mg/cig), Brand 5 yielded more differentially expressed genes than Brand 3 at the most active exposure condition (185 vs. 100 genes).

Although more genes were significantly differentially expressed following exposure to the Brand 1 CSC in our study, the overall gene expression analysis does not support the existence of a tobacco product-specific gene expression signature. By applying a large sample size, multiple concentrations and two time points, we found little evidence for CSC-specific profiles for the three brands analyzed. Although there were instances where fold changes may have been larger for certain brands (or have lower *P* values), the general trends among the brands were similar, with no concrete evidence that certain genes were responsive in a brand-specific manner. Clustering of all significant genes revealed that genes were grouped first by concentration, then time, with brand having little influence on the expression patterns. Using a more liberal analysis based on fold changes and unadjusted *P* values, and using RT-qPCR, we found that genes were highly correlated across the brands with no obvious brand-specific effects. Thus, we did not find evidence of brand specific signatures, despite differences in the way the brands are marketed.

The major effect of tobacco smoke on cell cycle observed in all studies is consistent with a p53-induced DNA damage response and chromosome damage at elevated concentrations. Indeed, disruption of cell cycle and oxidative stress are two pathways by which clastogenicity is thought to arise in cells exposed to CSC in vitro [DeMarini,^2004^; Guo et al.,^2006^,^2011^; Lu et al.,^2007^; DeMarini et al.,^2008^]. Cluster analyses on genes from these pathways (p53 and cell cycle) demonstrate that genes are correlated primarily by concentration, followed by time, and finally brand, which does not exert a major effect on gene expression, again supporting our conclusion that there are no clear brand-specific effects for the three CSCs examined in the present study.

The present work is also consistent with our earlier findings in vivo in lung samples collected from mice exposed for 6 or 12 weeks to mainstream tobacco smoke. Employing the same Agilent array and analytical methods, Halappanavar et al. [2009] found differential expression of 79 genes following chronic smoke exposure. Fifteen of these genes were differentially regulated in the same direction as in the present work on cultured FE1 epithelial cells, and included: *Cyp1a1*, *Cyp1b1*, *Ahr*, *Nqo1*, *Srxn1*, *Aldh3a1*, *Alk1*, *Gclm*, *Hmox1*, *Il6*, *Ptgs2*, *Ier3*, *Pdgfrb*, *Klf9*, and *Lincr*. A large number of commonalities in pathways and functions are also evident; including xenobiotic metabolism, redox balance, oxidative stress, glutathione metabolism, inflammatory response, heat shock proteins, signal transduction pathways, and transport (12 members of the solute carrier family were differentially regulated in vitro). Thus, despite noteworthy differences in the exposure regime (i.e., in vitro vs. in vivo), concentration, time, exposure material (i.e., mainstream tobacco smoke vs. CSC), we see a high degree of functional overlap in gene expression, which implies that the results obtained in vitro are relevant to in vivo outcomes.

## CONCLUSIONS

The present study confirms that CSC is clastogenic, cytotoxic, and mutagenic, with little differences in potencies across three CSCs representing three different tobacco products. The molecular pathways and biological functions affected by exposure to CSCs are consistent with previous studies demonstrating xenobiotic metabolism, oxidative stress, DNA damage response leading to cell cycle arrest and apoptosis, as well as inflammation. Moreover, at matched CSC concentrations, the brands examined, which included both full-flavor and light, and a blonde tobacco product, exhibited highly similar toxicogenomic responses and no evidence of brand-specific gene expression profiles. Thus, although the study only analyzed a small number of brands, the results provide no mechanistic support for any contention of harm reduction for a tobacco product marketed as “light.” It should be noted that we do not have quantitative information on smoking habits for the brands examined in this study, and thus, cannot comment on the risk of adverse health effects for the brands examined. However, it is well established that smokers adjust their smoking behavior to compensate for differences in nicotine content [Rickert and Robinson,^1981^; Kabat,^2003^; Benowitz et al.,^2005^; Hammond et al.,^2005^].

The present data provide a profile of gene expression signatures across a range of cigarette varieties, at two doses and time points, which can subsequently be used as a baseline for the evaluation of new tobacco products, including novel products that may be perceived as reduced or modified risk tobacco products. Several regulatory agencies, including Health Canada and the US Food and Drug Administration, are currently soliciting expert input regarding the utility of various toxicity assessment methodologies to critically evaluate cigarette manufacturer's claims of reduced harm.
