# Supplementary figures and images for: Genetic toxicology and toxicogenomic analysis of three cigarette smoke condensates in vitro reveals few differences among full-flavor, blonde, and light products
Source: Environ Mol Mutagen. 2012 Mar 19;53(4):281–96. doi: 10.1002/em.21689 (PMC3350776; doi:10.1002/em.21689)

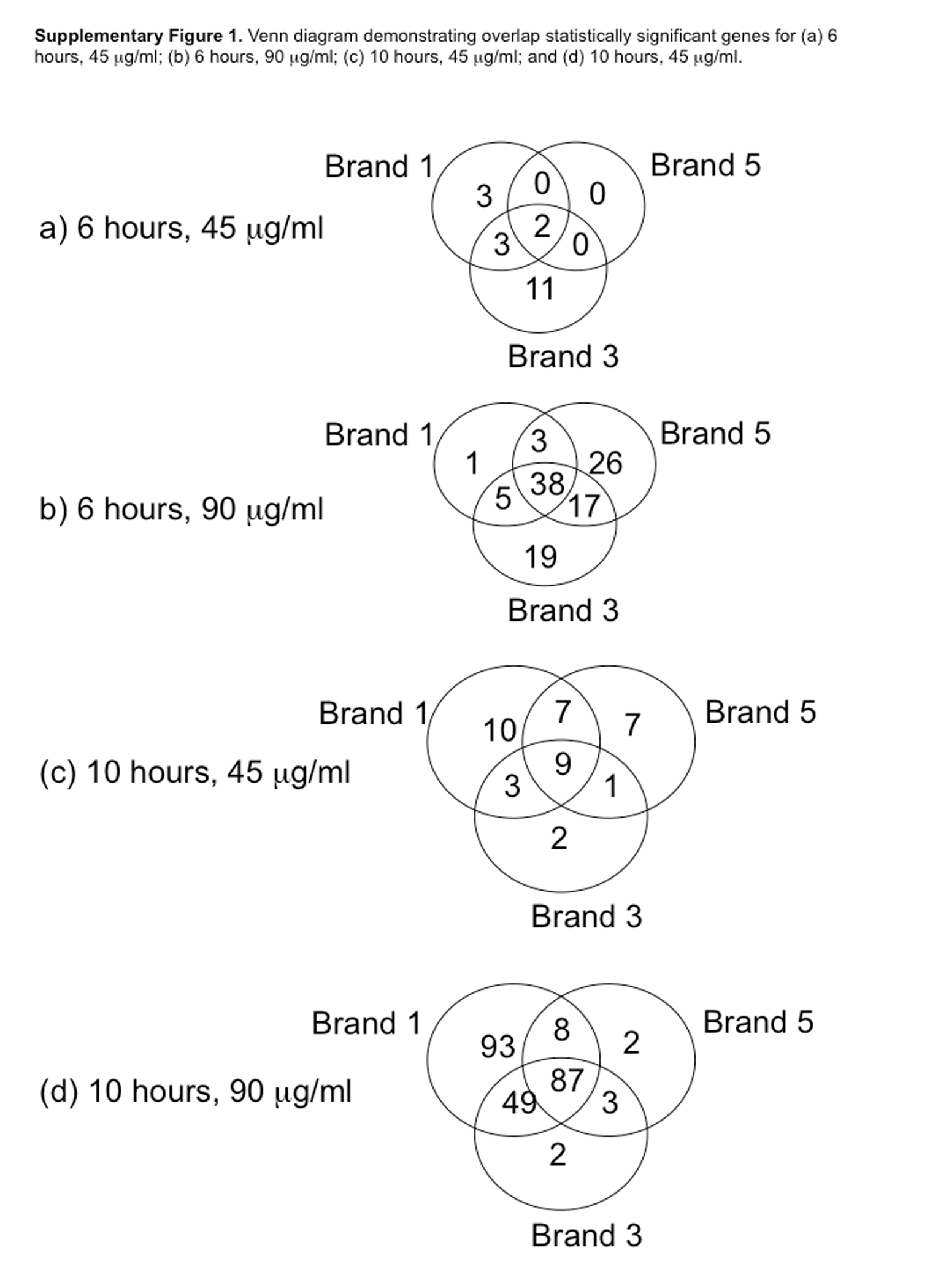

Supplement: Supplementary file 1 [file em0053-0281-SD1.tif]
